# A noninvasive model discriminating significant histological changes in treatment-naive chronic hepatitis B patients with normal ALT

**DOI:** 10.1186/s12985-023-01963-x

**Published:** 2023-01-11

**Authors:** Jiaming Teng, Yanan Du, Phimphone Visalath, Tianhui Zhou, Bingying Du, Qin Zhang, Wei Cai

**Affiliations:** 1 Department of Infectious Diseases, Ruijin Hospital, Shanghai Jiao Tong University School of Medicine, No 197, Ruijin 2nd Road, Shanghai, 200025 China; 2 Department of Infectious Diseases, Phase I Clinical Trial Unit, Tongren Hospital, Shanghai Jiao Tong University School of Medicine, 1111 XianXia Road, Shanghai, 200336 China

**Keywords:** Hepatitis B virus, Alanine aminotransferase, Liver stiffness, Liver histology

## Abstract

**Background:**

Traditionally part of chronic hepatitis B (CHB) patients with normal alanine aminotransferase (ALT) are recommended to antiviral therapy referring to liver biopsy. However, liver biopsy is an invasive method with various potential complications. A noninvasive model was established in the study to evaluate liver histology and to identify the need of antiviral therapy.

**Methods:**

A total of 614 liver biopsied CHB patients with ALT less than upper limit of normal from 2 centers were retrospectively analyzed. They were divided into a training cohort and a validation cohort. A noninvasive model to predict the significant liver histological changes was established and validated.

**Results:**

The results of analysis showed that ALT, Age, platelet (PLT) and liver stiffness (LS) were independent risk factors for significant liver injury. The model was established based on the 4 indexes, with the area under the curve of 0.85 and 0.87 in training cohort and validation cohort. Meanwhile, 2 cut-off scores were selected. By applying the low cut-off score (− 0.207), patients without significant liver injury could be identified with high accuracy, with negative predictive value of 72.7% and 73.7% in training and validation cohorts. By applying the high cut-off score (0.537), the presence of significant liver injury could be diagnosed with high accuracy, with positive predictive value of 90.3% and 88.8% in the training and validation cohorts. By applying the model, liver biopsy would have been avoided in 87.6% (538/614) patients, with correct prediction in 87.9% (473/538).

**Conclusion:**

The novel noninvasive model composed of ALT, Age, PLT, LS can correctly assess liver histology in CHB patient with normal ALT, which helps to determine the need of antiviral therapy without liver biopsy.

**Supplementary Information:**

The online version contains supplementary material available at 10.1186/s12985-023-01963-x.

## Background

There are more than 300 million people worldwide suffering from chronic hepatitis B virus (HBV) infection [1]. Furthermore, HBV is a leading cause of cirrhosis and hepatocellular carcinoma (HCC) [2]. In China, there are about 30 million patients infected with HBV [3]. Owing to the large population, eliminating HBV related liver diseases is still challenging [4].

Studies have shown that the leading causes of HBV progression are HBV replication and immune mediated liver damage. The persistent progression of inflammation and fibrosis can greatly increase the risk of cirrhosis and HCC in chronic hepatitis B (CHB) patients [5]. The existing antiviral drugs can effectively prevent inflammation and fibrosis progression, hepatic decompensation, and HCC development [6, 7]. Therefore, timely antiviral therapy is necessary for CHB patients. American Association for Liver Disease guidelines (2018) suggest that CHB patients with obvious inflammation or fibrosis, evaluated alanine aminotransferase (ALT) above 2 **×** upper limit of normal (ULN) (35 IU/ml in males and 25 IU/ml in females) and evaluated HBV DNA should receive antiviral therapy [8, 9]. European Association for the Study of Liver guidelines (2017) recommend that CHB patients with at least moderate inflammation or fibrosis, evaluated ALT above ULN (40 IU/ml) and detectable HBV DNA should receive antiviral therapy [10].

There is increasing evidence that CHB patients with normal ALT level could have significant liver injury, fulfilling histological indication for antiviral therapy [1]. Traditionally, the evaluation of liver inflammation and fibrosis in these patients is performed by liver biopsy. However, the invasive method is often not acceptable to patients [11]. Therefore, a noninvasive model to assess liver histology in CHB patients with normal ALT value is of vital importance.

Lots of noninvasive models, such as aspartate aminotransferase to platelet ratio index [12] and fibrosis index based on the four factors (FIB-4) [12, 13], were developed to assess liver histology. However, these models are established for various kinds of liver diseases. Their predictive value for identifying the need of antiviral therapy in CHB patients with normal ALT level is just mediocre.

In the study, a noninvasive model is established to predict liver histology in CHB patients with normal ALT value, and helps to determine whether such patients need antiviral therapy.

## Methods

### Patients

**Fig. 1:**
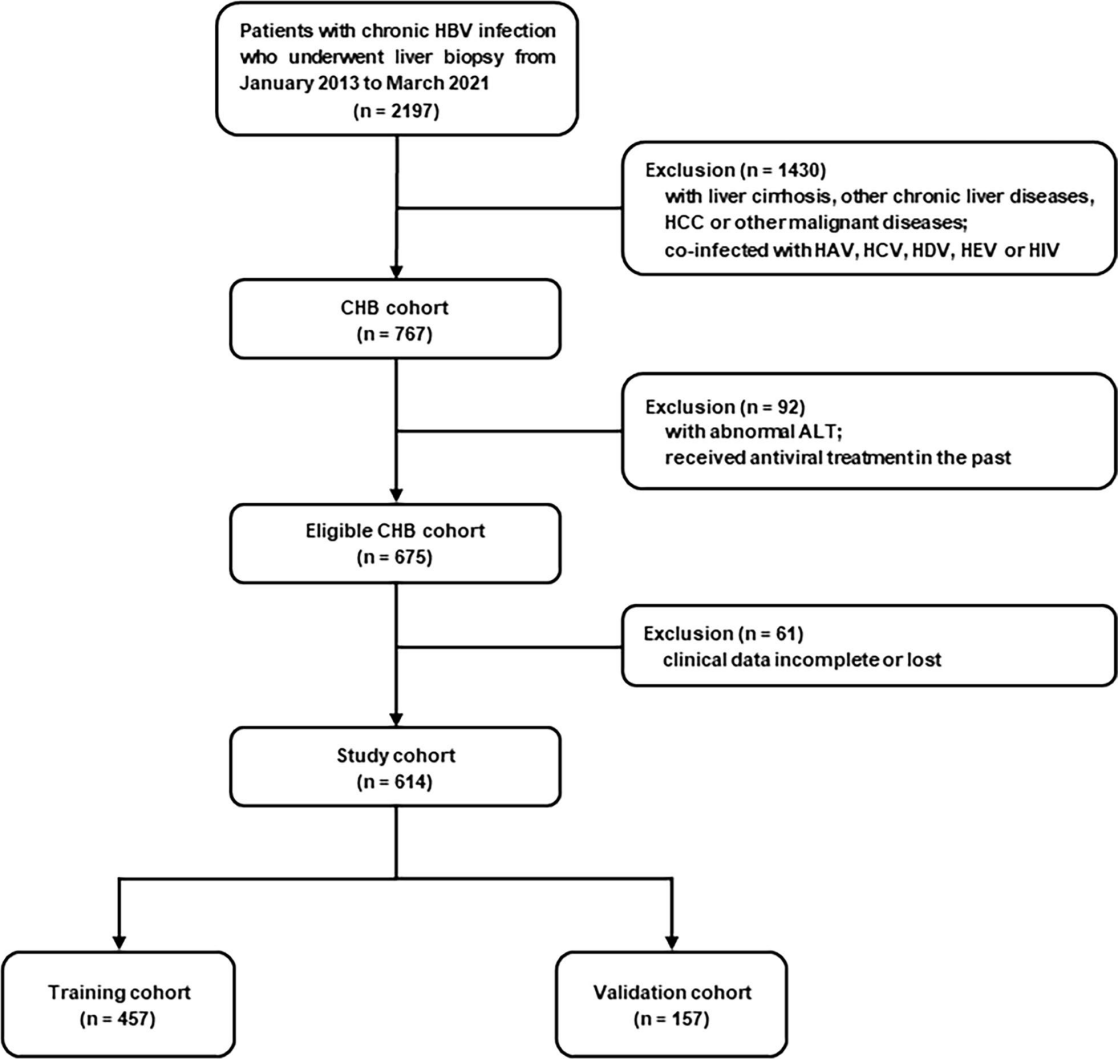
Inclusion and exclusion criteria for the CHB patients. *HBV* hepatitis B virus, *HCC* hepatocellular carcinoma, *ULN* upper limit of normal, *CHB* chronic hepatitis B, *ALT* alanine aminotransferase, *HAV* hepatitis A virus, *HCV* hepatitis C virus, *HDV* hepatitis D virus, *HEV* hepatitis E virus, *HIV* human immunodeficiency virus

A total of 614 CHB patients with normal ALT level who underwent liver biopsy from January 2013 to March 2021 in Ruijin Hospital and Tongren Hospital were included. The clinical data were retrospectively analyzed and all the cases without family history of cirrhosis and HCC. The inclusion criteria including the diagnosis of CHB, treatment-naïve CHB patients, serum ALT level < ULN (64 U/L) [14], detectable HBV DNA level and a liver biopsy with liver histology consistent with chronic HBV. Exclusion criteria including co-infection of HBV and other virus, alcohol liver disease, drug induced liver injury disease, autoimmune liver disease and nonalcoholic fatty liver disease, etc. [15] (Fig. 1). The study was conducted in accordance with the ethical guidelines of the Declaration of Helsinki Istanbul. The study was approved by the Human Ethics Committee of Ruijin Hospital (No. 2021-150) and Tongren Hospital (No. 2022-063-01). Written informed consent was waived by the Committee considering the retrospective design.

### Liver biopsy

Liver biopsy was performed through ultrasound-guided percutaneous using 16G needle. The liver specimens were obtained, fixed in formalin, embedded in paraffin. Hematoxylin–eosin–safran, and Masson’s trichrome were conducted according to the protocols to confirm the inflammation grade and fibrosis stage. The histologic grading of necroinflammation (G0–G4) and staging of liver fibrosis (S0–S4) were performed according to the Scheuer scoring system [16]. According to the results of histopathology, G ≥ 2 was considered moderate to severe inflammation, and S ≥ 2 was considered significant fibrosis. From this, significant liver injury was defined as G ≥ 2 and/or S ≥ 2, as histological indication for antiviral therapy.

### Clinical data of patients

Demographic and biochemical data of patients were collected within 2 weeks before liver biopsy, including age, sex, height, weight, HBV DNA, hepatitis B surface antigen (HBsAg), hepatitis B e antigen (HBeAg), hepatitis B e antibody (HBeAb), liver stiffness (LS), alpha fetoprotein (AFP), gamma-glutamyl transpeptidase (GGT), total bilirubin, total protein, bile acid, prealbumin; albumin, alkaline phosphatase, ALT, aspartate aminotransferase (AST), direct bilirubin, prothrombin time, international normalized ratio and platelet (PLT).

### Study design and statistical analysis

The main endpoint of the study was to identify the need of antiviral therapy of CHB patients by a combination of relevant variables. In the study, 457 cases and 157 cases were randomly divided into a training cohort and a validation cohort. The data of 457 patients were used to establish the prediction model, and the data of 157 patients were used to validate the prediction model. Quantitative variables were expressed as the mean **±** standard deviation (SD), which were compared with Student t test or Wilcoxon test. Categorical variables were demonstrated with number percentage and were compared using Chi-squared method. Univariate and multivariate logistic regression analyses were used to evaluate the independent variables for the indication for antiviral therapy. The variables with *p* < 0.05 by multivariate analysis were used to construct a scoring system to predict the moderate to severe inflammation or significant fibrosis. Performance of the noninvasive model was analyzed by the receiver operator characteristic (ROC) curve. The area under the ROC curve (AUROC) was evaluated, and the cut-off value was determined by the ROC curve at the maximum Jordan index. The efficacy of the noninvasive model was evaluated by specificity, sensitivity, positive predictive value (PPV), negative predictive value (NPV), precision, and recall. Finally, a high cut-off value and a low cut-off value were determined to get the ideal PPV and NPV for the moderate to severe inflammation or significant fibrosis. All *p* values are two-sided, and *p* < 0.05 was considered to be statistically significant. Statistical analyses were performed using R i368 4.0.5.

## Results

### Characteristics of patients

The clinical data including age, height, weight and other laboratory indexes of all the patients, as well as the training cohort and validation cohort were shown in Table 1. There were 457 cases in the training cohort and 157 cases in the validation cohort. A total of 364 patients in the training cohort and 117 in the validation cohort were diagnosed as significant liver injury by liver biopsy (Table 1). In the training cohort, 364 (79.6%) patients needed antiviral therapy. In the validation cohort, 117 (74.5%) patients needed antiviral therapy (Table 1). Besides, there were significant differences in age, LS, ALT and PLT between subgroups in both training and validation cohorts (Table 1).

**Table 1:** Characteristics of the enrolled cases

Variables at baseline	Total cohort (n = 614)	Training cohort (n = 457)				Validation cohort (n = 157)			
		G < 2 and S < 2 (n = 93)	G ≥ 2 and/or S ≥ 2 (n = 364)	*p* value*	Total	G < 2 and S < 2 (n = 40)	G ≥ 2 and/or S ≥ 2 (n = 117)	*p* value^#^	Total
Age (years)	41.0 (10.7)	38.3 (10.7)	41.9 (11.2)	0.02	41.2 (11.1)	39.6 (11.2)	40.79 (10.5)	0.04	40.3 (10.6)
Male gender, n (%)	422 (68.7%)	63 (67.7%)	252 (69.2%)	0.88	315 (68.9%)	27 (67.5%)	80 (68.4%)	0.99	107 (68.2%)
Height (m)	168.3 (7.5)	168.4 (7.4)	168.9 (7.5)	0.40	168.4 (7.5)	167.9 (6.5)	168.7 (7.8)	0.57	168.3 (7.4)
Weight (kg)	67.9 (12.0)	67.8 (10.5)	68.1 (12.4)	0.10	67.7 (12.2)	65.2 (12.3)	67.9 (11.9)	0.09	67.2 (12.2)
HBV DNA (log_10_IU/ml)	5.0 (2.2)	5.0 (2.5)	5.14 (2.0)	0.73	4.9 (2.1)	5.0 (2.6)	5.1 (2.0)	0.79	5.1 (2.0)
LS (kPa)	8.2 (4.1)	5.5 (1.5)	8.9 (4.4)	0.0001	8.3 (4.3)	4.5 (1.3)	8.9 (5.0)	0.0001	7.8 (4.6)
AFP (ng/mL)	5.2 (5.3)	5.1 (2.5)	5.9 (16.6)	0.26	5.4 (69.9)	4.7 (1.8)	4.8 (2.2)	0.31	4.8 (6.3)
GGT (IU/L)	30.8 (17.1)	31.3 (16.7)	32.4 (36.1)	0.71	31.0 (44.9)	30.3 (20.1)	30.0 (18.4)	0.62	30.1 (22.8)
TBIL (µmol/L)	16.4 (8.7)	16.9 (6.7)	17.1 (10.7)	0.05	16.2 (11.8)	15.9 (8.3)	17.2 (8.6)	0.16	16.8 (8.5)
TP (mg/L)	73.3 (7.2)	72.6 (4.6)	73.2 (7.9)	0.08	73.3 (7.0)	73.6 (4.6)	73.15 (7.9)	0.59	73.2 (7.2)
BA (µmol/L)	8.7 (13.1)	8.1 (30.6)	8.3 (11.5)	0.39	8.7 (14.7)	8.5 (21.5)	8.6 (19.2)	0.94	8.6 (8.4)
PAB (mg/L)	224.0 (48.5)	214.7 (51.6)	221.1 (48.2)	0.12	225.3 (49.6)	229.8 (46.9)	217.1 (49.8)	0.12	220.4 (49.2)
ALB (g/l)	44.4 (4.5)	45 (3.6)	44.9 (4.6)	0.40	44.5 (4.5)	44.4 (3.2)	44.05 (4.4)	0.81	44.1 (4.2)
AKP (IU/L)	74.6 (22.8)	73.0 (23.9)	75.0 (18.7)	0.06	74.8 (27.9)	73.0 (23.1)	73.5 (26.2)	0.18	73.4 (20.4)
ALT (IU/L)	43.2 (24.4)	29.7 (14.5)	48.3 (29.5)	0.0001	44.7 (14.3)	27.53 (13.2)	43.6 (18.4)	0.001	39.0 (14.5)
AST (IU/L)	35.2 (18.9)	30.6 (20.9)	35.7 (17.1)	0.05	34.7 (20.2)	32.4 (21.2)	35.6 (19.8)	0.07	36.8 (32.9)
DBIL (µmol/L)	3.1 (2.1)	3.1 (1.3)	3.2 (3.2)	0.07	3.2 (3.5)	2.7 (1.2)	3.1 (2.0)	0.18	3.0 (1.85)
PT (s)	11.7 (1.1)	11.5 (0.8)	11.8 (0.9)	0.32	11.7 (1.0)	11.6 (0.8)	11.7 (0.9)	0.71	11.7 (0.9)
INR	1.0 (0.5)	0.9 (0.1)	1.0 (0.1)	0.06	1.0 (0.1)	1.0 (0.7)	1.0 (0.7)	0.68	1.0 (0.7)
PLT (10^9^/L)	179.6 (51.1)	183.9 (46.1)	176.6 (50.7)	0.03	178.1 (49.1)	199.6 (51.8)	179.7 (53.0)	0.04	183.9 (53.4)
HBeAg+, n (%)	348 (56.7%)	51 (54.8%)	211 (58.0%)	0.67	262 (57.3%)	22 (55.0%)	64 (54.7%)	0.99	86 (54.8%)

The table shows the mean (SD) for continuous variables and number (%) for binary variables

*HBV* hepatitis B virus, *LS* liver stiffness, *AFP* alpha fetoprotein, *HBeAg* hepatitis B e antigen, *GGT* gamma-glutamyl transpeptidase, *TBIL* total bilirubin, *TP* total protein, *BA* bile acid, *ALB* albumin, *PAB* prealbumin, *AKP* alkaline phosphatase, *ALT* alanine transaminase, *AST* aspartate aminotransferase, *DBIL* direct bilirubin, *PT* prothrombin time, *INR* international normalized ratio, *PLT* platelet

*Difference of data between 2 subgroups in training cohort

^#^Difference of data between 2 subgroups in validation cohort

### Predictors of the significant liver injury

To investigate the risk factors for significant liver injury in CHB patients with normal ALT, the clinical data were analyzed by univariate and multivariate analyses. The results in the training cohort and the validation cohort were shown in Table 2. Multivariate analysis revealed that ALT, Age, LS, and PLT were significantly associated with significant liver injury.

**Table 2:** Variables associated with the significant liver injury in training cohort

Variables	Univariate analysis			Multivariate analysis		
	OR	95%CI	*p* value	OR	95%CI	*p* value
Age (years)	1.03	1.01–1.05	0.01	1.04	1.01–1.07	0.005
Height (m)	1.01	0.94–1.04	0.73			
Weight (kg)	1.02	0.99–1.04	0.14			
HBV DNA (log_10_IU/ml)	1.55	1.06–1.98	0.14			
LS (kPa)	1.88	1.61–2.25	< 0.0001	1.77	1.44–2.06	< 0.0001
AFP (ng/mL)	1.13	1.03–1.29	0.03			
GGT (IU/L)	1.03	0.99–1.06	0.08			
TBIL (µmol/L)	1.03	1.00-1.09	0.11			
TP (mg/L)	1.02	0.98–1.05	0.32			
BA (µmol/L)	0.99	0.98–1.16	0.64			
PAB (mg/L)	0.98	0.97–0.99	0.13			
ALB (g/l)	0.89	0.83–1.04	0.71			
AKP (IU/L)	1.00	0.99–1.02	0.53			
ALT (IU/L)	1.06	1.04–1.08	0.001	1.04	1.03–1.09	< 0.0001
AST (IU/L)	1.09	1.06–1.14	0.02			
DBIL (µmol/L)	1.26	1.04–1.55	0.08			
PT (s)	1.75	1.30–2.39	0.19			
INR	2.01	0.90–3.11	0.37			
PLT (10^9^/L)	1.02	1.01–1.06	0.04	1.01	1.01–1.02	0.02

*HBV* hepatitis B virus, *LS* liver stiffness, *AFP* alpha fetoprotein, *GGT* gamma-glutamyl transpeptidase, *TBIL* total bilirubin, *TP* total protein, *BA* bile acid, *ALB* albumin, *PAB* prealbumin, *AKP* alkaline phosphatase, *ALT* alanine transaminase, *AST* aspartate aminotransferase, *DBIL* direct bilirubin, *PT* prothrombin time, *INR* international normalized ratio, *PLT* platelet

### Model building

**Fig. 2:**
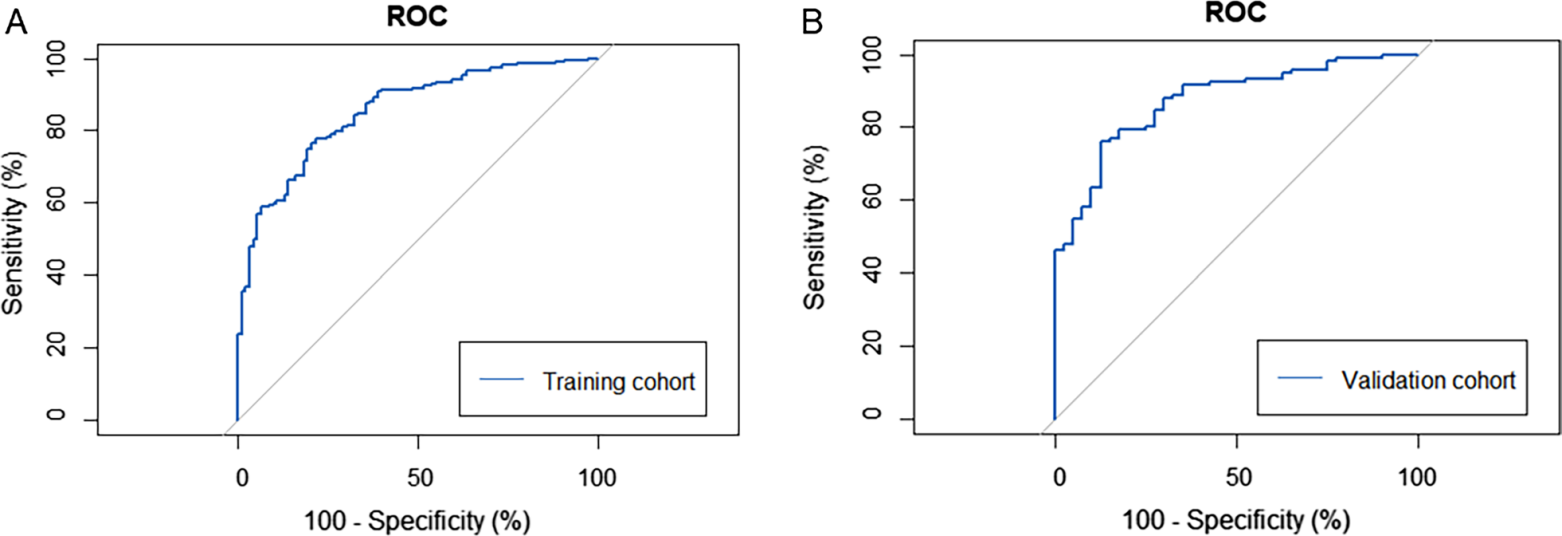
ROC curve of the novel model for identifying significant liver injury in training cohort and validation cohort. **A** ROC curve of the scoring system in the training cohort (n = 457) combining 4 variables (Age, LF, ALT, PLT) to distinguish between CHB patients with and without significant liver injury. The area under the ROC curve is 0.85. **B** ROC curve of the scoring system in the validation cohort (n = 157) combining 4 variables (Age, LF, ALT, PLT) to distinguish between CHB patients with and without significant liver injury. The area under the ROC curve is 0.87. *ROC* receiver operator characteristic, *LF* liver stiffness, *ALT* alanine aminotransferase, *PLT* platelet, *CHB* chronic hepatitis B

Based on the risk factors for significant liver injury, a novel index was developed: AAPL index **=** − 6.5191 + 0.5724 × LS (kPa) + 0.0381 × ALT (IU/L) + 0.0388 × Age (year) + 0.0068 × PLT (10^9^/L). The AUROC of the noninvasive model was 0.85 for identification of significant liver injury in the training cohort (Fig. 2A).

Based on the ROC curve, 2 cut-off points were identified to differentiate patients with significant liver injury from those without. By applying the low cut-off value (− 0.207), 32 of 44 CHB patients with normal ALT who didn’t need antiviral therapy immediately were correctly identified. The NPV was 72.7% when using the low cut-off value. By applying the high cut-off value (0.537), 324 of 359 CHB patients with normal ALT who needed antiviral therapy were correctly identified (Table 3). The PPV was 90.3% when using the high cut-off value.

**Table 3:** Predictive value of the scoring system for significant liver injury in CHB patients with normal ALT levels in training cohort

	AAPL index			Total
	< low cut-off (< − 0.207)	Indeterminate (− 0.207 to 0.537)	> high cut-off (> 0.537)	
Total	44	54	359	457
G < 2 and S < 2	32	26	35	93
G ≥ 2 or/and S ≥ 2	12	28	324	364
Specificity (%)	34.4		62.4	
Sensitivity (%)	96.7		89.0	
PPV (%)	85.2		90.3	
NPV (%)	72.7		59.2	
Correct prediction rate	(324 + 32)/(359 + 44) = 88.3%			

A total of 403 (359 + 44) patients were identified by AAPL index, in detail, 359 with and 44 without significant liver injury. Among them, the prediction of 356 (324 + 32) patients were proven correct by hepatic histopathology

*CHB* chronic hepatitis B, *G* grading of necroinflammation, *S* staging of liver fibrosis, *PPV* positive predictive value, *NPV* negative predictive value

 Overall, in the training cohort, 359 patients were identified with significant liver injury, and 44 patients were identified without significant liver injury by AAPL scores, with a total number of 403 (359 + 44). Among these identified patients, 324 patients with significant liver injury and 32 patients without significant liver injury were correctly diagnosed, with a total number of 356 (324 + 32). Thus, by applying the model to the training cohort, a liver biopsy would have been avoided in 88.2% (403/457) patients, with correct prediction rate of 88.3% (356/403) (Table 3).

### Model validation

In the validation cohort, the AUROC of the noninvasive model was 0.87 (Fig. 2B), which also showed a good performance. By applying the low cut-off value (− 0.207), 14 of 19 CHB patients with normal ALT who didn’t need antiviral therapy immediately were correctly identified. The NPV was 73.7% when using the low cut-off value. By applying the high cut-off value (0.537), 103 of 116 CHB patients with normal ALT who needed antiviral therapy were correctly identified. The PPV was 88.8% when using the high cut-off value (Table 4).

**Table 4:** – Predictive value of the scoring system for significant liver injury in CHB patients with normal ALT levels in validation cohort

	AAPL index			Total
	< low cut-off (< − 0.207)	Indeterminate (− 0.207 to 0.537)	> high cut-off (> 0.537)	
Total	19	22	116	157
G < 2 and S < 2	14	13	13	40
G ≥ 2 or/and S ≥ 2	5	9	103	117
Specificity (%)	35.0		67.5	
Sensitivity (%)	95.7		88.0	
PPV (%)	81.2		88.8	
NPV (%)	73.7		65.9	
Correct prediction rate	(103 + 14)/(116 + 19) = 86.7%			

A total of 135 (116 + 19) patients were identified by AAPL index, in detail, 116 with and 19 without significant liver injury. Among them, the prediction of 117 (103 + 14) patients were proven correct by hepatic histopathology

*CHB* chronic hepatitis B, *G* grading of necroinflammation, *S* staging of liver fibrosis, *PPV* positive predictive value, *NPV* negative predictive value

 Overall, in the validation cohort, 116 patients were identified with significant liver injury, and 19 patients were identified without significant liver injury by AAPL scores, with a total number of 135 (116 + 19). Among these identified patients, 103 patients with significant liver injury and 14 patients without significant liver injury were correctly diagnosed, with a total number of 117 (103 + 14). Thus, by applying the model to the validation cohort, a liver biopsy would have been avoided in 86.0% (135/157) patients, with correct prediction rate of 86.7% (117/135) (Table 4).

### Predictive value of various noninvasive methods for antiviral therapy decision in patients with CHB

In the training cohort, the AUROCs of ALT, LS, Age and PLT for antiviral therapy decision were 0.762, 0.795, 0.585 and 0.561, respectively (Table 5). The noninvasive model, which combined ALT, LS, Age and PLT, showed better performance with the AUROC of 0.851. Furthermore, performances of the most common noninvasive models, APRI and FIB-4, were unsatisfactory with the AUROCs of 0.631 and 0.623 (Table 5). Accordingly, the AAPL model is a more reliable noninvasive method to identify the need of antiviral therapy than single variable and other noninvasive models.

**Table 5:** Predictive value of various noninvasive methods to identify histological indication for antiviral therapy in training cohort

	AUROC	95%CI
APRI	0.631	0.561–0.687
FIB-4	0.623	0.567–0.691
ALT	0.762	0.709–0.814
LS	0.795	0.760–0.852
Age	0.585	0.520–0.650
PLT	0.561	0.532–0.607
AAPL model	0.851	0.814–0.894

*CHB* chronic hepatitis B, *APRI* aspartate aminotransferase to platelet ratio index, *FIB-4* fibrosis index based on the four factors, *ALT* alanine transaminase, *LS* liver stiffness, *PLT* platelet, *AUROC* area under the receiver operator characteristic curve, *CI* confidence interval

### Clinical efficacy of antiviral therapy

 We further analyzed the prognosis in patients identified with significant liver injury needing antiviral therapy by the model, and patients followed up for more than 1 year were included. Among them, 356 patients initiated antiviral therapy after liver biopsy were included in therapy group, and 22 patients followed up with no antiviral therapy were included in non-therapy group. In the therapy group, 165 (97.1%) patients achieved complete virological response and 5 (2.9%) patients achieved HBeAg seroconversion. While no patients in non-therapy group achieved complete virological response or HBeAg seroconversion. Besides, no patients in therapy or non-therapy group achieved HBsAg seroconversion, or developed cirrhosis or HCC (Additional file 1: Table S1). It was indicated that those patients with normal ALT who were identified by the model as need antiviral therapy benefited from the antiviral therapy in the real world, and had better prognosis than those were not treated.

## Discussion

In the study, we established and validated a noninvasive model named AAPL which were composed of easily available laboratory and clinical indexes. The model can be used to identify the absence or presence of significant liver injury in CHB patients with normal ALT level, and to evaluate whether this part of patients need antiviral therapy immediately.

CHB patients have an increased risk of liver cirrhosis and HCC [17]. Early control of HBV activity can prevent the occurrence of end-stage liver diseases [9, 18]. Studies have shown that antiviral therapy can promote HBeAg and HBsAg seroconversion. It is reported that HBeAg is important in the history of chronic HBV infection. During the course of HBV infection, HBeAg seroconversion often coincides with normalization of biochemical test and clinical remission [19–21]. There is a lower incidence of cirrhosis and HCC among patients who undergo spontaneous or treatment-induced HBeAg seroconversion [21, 22]. HBeAg seroconversion is also associated with a higher probability of HBsAg loss and seroconversion, which is considered to be associated with clinical cure of HBV infection [21, 23]. All CHB patients meeting the antiviral therapy indication should get treated in order to prevent the progression of liver injury [10].

Based on the current guidelines, HBeAg positive patients with normal ALT level and high HBV viral load, as well as HBeAg negative with normal ALT level and low HBV viral load are required regular follow-up [9, 10]. There is a proportion of CHB patients with normal ALT level having significant liver injury, who should get antiviral therapy immediately. Traditionally, such patients are identified after liver biopsy. However, liver biopsy, as an invasive examination with a series of potential complications is often not acceptable to patients [11, 24]. Thus, it is challenging to identify CHB patients with normal ALT level who need antiviral therapy. So, we establish a noninvasive prediction model to solve this problem.

It has been showed in previous studies that ALT, Age, PLT and LS were independent variables for severity of liver injury [25–27]. In the study, we also found that there are positive correlations between these indexes and liver injury, in agreement with the previous reports. Furthermore, we developed the prediction model based on these 4 indexes. Our noninvasive model proved to have satisfactory diagnostic value, which helps to identify those who need initiating antiviral therapy immediately among CHB patients with normal ALT level.

Most noninvasive models only focus on predicting the stage of liver fibrosis or the grade of the liver inflammation. However, according to clinical guidelines on HBV infection nowadays, we should take both fibrosis stage and inflammation grade into account. A noninvasive model composed of PT, AST, GGT, and anti-hepatitis B core antibody levels is valuable in predicting significant liver inflammation in CHB patients [28], however, it ignores the fibrosis stage. Conversely, another noninvasive model called CPHBV could accurately predict liver fibrosis in CHB patients with normal ALT level, regardless of liver inflammation [29]. AGH model composed of AST, hepatitis B core antigen and GGT is a reliable index to predict moderate to severe inflammation or significant fibrosis, which helps to determine the initiation of antiviral therapy [30]. But the predictive value of our model is better than AGH model.

Based on the logistic analysis, we establish a noninvasive model named AAPL, which combined ALT, LS, Age and PLT. According to the ROC curve, the AUCs in the training cohort and the validation cohort were 0.85 and 0.87, both satisfactory. Furthermore, for CHB patient with normal ALT level, we recommend antiviral therapy when AAPL index is above the high cut-off value, and regular monitoring when it is below the low cut-off value. Or rather, patients whose AAPL index is beyond the 2 cut-off values could avoid liver biopsy.

The treatment of CHB was mainly based on serum HBV DNA level, serum ALT level and severity of hepatic inflammation and fibrosis according to the guidelines [31, 32]. Previous studies suggested that LS, Age and PLT were risk factors for liver injury in patients with chronic HBV infection [25, 26]. In our study, logistics analysis showed that ALT level, LS, Age and PLT were independent factors for liver injury. Our findings were consistent with clinical guidelines and previous studies, which indicated the reliability of the model.

Our study has several advantages. It included a relatively large cohort of treatment-naïve CHB patients with normal ALT level. The research is a multicenter study, and the noninvasive model has been validated in a cohort from 2 centers. The newly established model consists of clinical and laboratory variables readily available during regular follow-up of CHB in clinical practice. It is easy for patients to use, and has good performance in the identification of antiviral therapy. Treatment-naïve CHB patients could benefit from the model to assess their hepatic histology during their follow-up, and to start their antiviral therapy on time without liver biopsy.

### Conclusions

In conclusion, a proportion of CHB patients with normal ALT level, who proves to have significant liver injury referring to liver biopsy, should receive antiviral therapy. ALT level, LS, Age and PLT were independent variables to identify significant liver injury. The noninvasive model consisting of the 4 indexes, can be used to identify histological indication for antiviral therapy, which helps to reduce the need of liver biopsy.

## Supplementary Information

**Additional file 1: Table S1.** Prognosis of CHB patients identified with significant liver injury by AAPL index.

## Data Availability

The datasets generated and/or analyzed during the current study are not publicly available but are available from the corresponding author on reasonable request.

## Abbreviations

HBV: Hepatitis B virus
HCC: Hepatocellular carcinoma
CHB: Chronic hepatitis B
ALT: Alanine aminotransferase
ULN: Upper limit of normal
HBsAg: Hepatitis B surface antigen
HBeAg: Hepatitis B e antigen
HBeAb: Hepatitis B e antibody
LS: Liver stiffness
GGT: Gamma-glutamyl transpeptidase
AST: Aspartate aminotransferase
PLT: Platelet
ROC: Receiver operator characteristic
PPV: Positive predictive value
NPV: Negative predictive value
