# Supplementary material for: A noninvasive model discriminating significant histological changes in treatment-naive chronic hepatitis B patients with normal ALT
Source: Virol J. 2023 Jan 11;20:7. doi: 10.1186/s12985-023-01963-x (PMC9835358; doi:10.1186/s12985-023-01963-x)
Supplement: Supplementary file 1 — Additional file 1: Table S1. Prognosis of CHB patients identified with significant liver injury by AAPL index. [file 12985_2023_1963_MOESM1_ESM.doc]

**Additional file 1: Table S1– Prognosis of CHB patients identified with significant liver injury by AAPL index**

| **Variables** | **Therapy group**  **(n=356)** | **Non-therapy group**  **(n=22)** |
| --- | --- | --- |
| Complete virological response^#^ (%) | 165 (97.1%) | 0 (0) |
| HBeAg seroconversion (%^*^) | 5 (2.9%) | 0 (0) |
| HBsAg seroconversion (%) | 0 (0) | 0 (0) |
| Cirrhosis (%) | 0 (0) | 0 (0) |
| HCC (%) | 0 (0) | 0 (0) |

^#^ Complete virological response is defined as serum HBV DNA level < 500 IU/mL.

^*^ The percentages of HBeAg seroconversion were based on 170 and 13 patients with positive HBeAg at baseline.

Abbreviations: HBV, hepatitis B virus; HBsAg, hepatitis B surface antigen; HBeAg, hepatitis Be Antigen; HCC, hepatocellular carcinoma
